# Inhibition of SARS-CoV-2 3CL protease by the anti-viral chimeric protein RetroMAD1

**DOI:** 10.1038/s41598-023-47511-z

**Published:** 2023-11-17

**Authors:** Lee-Chin Chan, Aini Syahida Mat Yassim, Abdullah Al Hadi Ahmad Fuaad, Thean Chor Leow, Suriana Sabri, Radin Shafierul Radin Yahaya, Awang Muhammad Sagaf Abu Bakar

**Affiliations:** 1 Biovalence Sdn. Bhd., 22, Jalan SS 25/34, Taman Mayang, 47301 Petaling Jaya, Selangor Malaysia; 2 Biovalence Technologies Pte. Ltd., #06-307 The Plaza, 7500A Beach Road, Singapore, 199591 Singapore; 3 School of Health Science, Universiti Sains Malaysia, 16150 Kubang Kerian, Kelantan, Malaysia; 4 Centre of Fundamental and Frontier Sciences in Self-Assembly (FSSA), Department of Chemistry, Faculty of Science, Universiti Malaya, 50603 Kuala Lumpur, Malaysia; 5 Department of Microbiology, Faculty of Biotechnology and Biomolecular Sciences, Universiti Putra Malaysia, 43400 UPM Serdang, Selangor Malaysia; 6 Enzyme and Microbial Technology Research Center, Faculty of Biotechnology and Biomolecular Sciences, Universiti Putra Malaysia, 43400 UPM Serdang, Selangor Malaysia; 7 Institute of Bioscience, Universiti Putra Malaysia, 43400 UPM Serdang, Selangor Malaysia; 8 Jabatan Perkhidmatan Veterinar Sabah, Aras 3, Blok B, Wisma Pertanian Sabah, Jalan Tasik, Luyang (Off Jln Maktab Gaya), Beg Berkunci 2051, 88999 Kota Kinabalu, Sabah Malaysia

**Keywords:** Biotechnology, Computational biology and bioinformatics, Drug discovery, Diseases, Health care

## Abstract

COVID-19 results from SARS-CoV-2, which mutates frequently, challenging current treatments. Therefore, it is critical to develop new therapeutic drugs against this disease. This study explores the interaction between SARS-CoV-2 3CL^pro^ and RetroMAD1, a well-characterized coronavirus protein and potential drug target, using in-silico methods. The analysis through the HDOCK server showed stable complex formation with a binding energy of -12.3, the lowest among reference drugs. The RetroMAD1-3CL^pro^ complex underwent a 100 ns molecular dynamics simulation (MDS) in an explicit solvation system, generating various trajectories, including RMSD, RMSF, hydrogen bonding, radius of gyration, and ligand binding energy. MDS results confirmed intact interactions within the RetroMAD1-3CL^pro^ complex during simulations. In vitro experiments validated RetroMAD1's ability to inhibit 3CL^pro^ enzyme activity and prevent SARS-CoV-2 infection in human bronchial cells. RetroMAD1 exhibited antiviral efficacy comparable to Remdesivir without cytotoxicity at effective concentrations. These results suggest RetroMAD1 as a potential drug candidate against SARS-CoV-2, warranting further in vivo and clinical studies to assess its efficiency.

## Introduction

In late 2019, an outbreak of pneumonia with an unknown origin emerged in Wuhan, China. Subsequently, it rapidly spread worldwide, placing significant burdens on global public healthcare and economic systems. The outbreak was later attributed to a coronavirus, named severe acute respiratory syndrome coronavirus 2 (SARS-CoV-2), which resulted in the disease being termed coronavirus disease (COVID-19)^1^. As of 6 September 2023, SARS-CoV-2 has caused 770 million confirmed cases and 6.9 million deaths globally according to the World Health Organisation (WHO) (https://covid19.who.int/data, 6 September 2023). This pandemic has prompted researchers worldwide to join forces, accelerating innovation to combat COVID-19 effectively. Since then, multiple vaccine candidates have entered clinical trials. The large-scale distribution of COVID-19 vaccines that began in early 2021 marked a significant turning point in the global battle against the pandemic. In May 2023, the WHO downgraded the COVID-19 pandemic status and announced that it was no longer a global public health emergency^2^.

**Figure 1:**
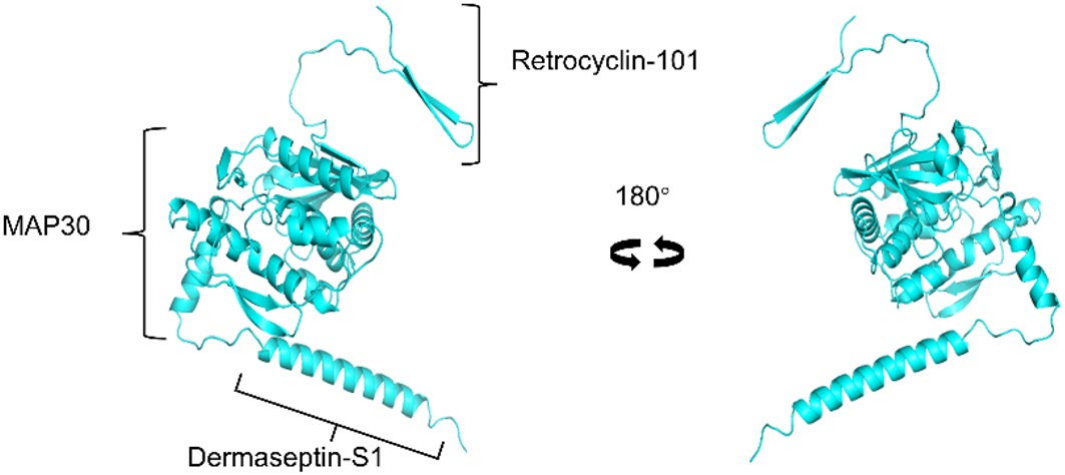
3D Structural Model of RetroMAD1. The predicted structure consists of complete domain of Retrocyclin- 101, MAP-30 and Dermaseptin S-1 model.

Despite most countries transitioning to endemic phases, the continuous emergence of new SARS-CoV-2 variants makes COVID-19 an ongoing concern^3,4^. To prepare for future waves of infection, it is crucial to develop vaccines and new drugs using various platform technologies. In the race to combat COVID-19, we introduce our chimeric protein, RetroMAD1 (Patent Number: US 2013/0336955A1). RetroMAD1, when administered orally, has demonstrated effectiveness against the Feline Leukemia Virus (FeLV), a pathogen in cats^5^. Comprising 41.3 kDa, RetroMAD1 is a combination of three different antimicrobial proteins: Retrocyclin-101 linked to the N-terminus of the 30 kDa momordica anti-human immunodeficiency virus (HIV) protein (MAP-30) via a linker peptide, with Dermaseptin-S1 directly joined to MAP-30 at the C-terminus (Fig. 1)^6^.

Retrocyclins are stable 18-residue defensin peptides characterised by their cationic nature and β-sheet structure, known for their resilience to boiling, acidic conditions, and other harsh environments^7,8^. Retrocyclin-101, a non-hemolytic and minimally cytotoxic variant, exhibits potent antiviral activity against HIV-1 by binding to gp120 with 25-fold greater affinity than retrocyclin^9,10^. Furthermore, retrocyclin-101 has shown efficacy against flaviviruses, influenza viruses, bacterial vaginosis, *Staphylococcus aureus*, Herpes Simplex viruses, and SARS-CoV-2 through various mechanisms^11–16^.

MAP-30 is a single stranded protein with 263-residue derived from *Momordica charantia* seeds, possesses anti-HIV and anti-tumour properties and inhibits the replication of Herpes Simplex Virus^17–25^. Belonging to the family of type-1 ribosomal inactivating proteins (RIPs), MAP-30 exhibits N-glycosidase activity that inactivates ribosomes, thereby inhibiting protein synthesis^26–29^. Additionally, MAP-30 can disrupt viral DNA and ribosomal function in virally infected cells, providing novel pathways for its antiviral activity^24^.

Dermaseptin-S1, a 34-residue antimicrobial defensin peptide, was initially purified from the skin extract of the South American arboreal frog *Phyllomedusa Sauuagii* by Mor et al. in 1991^30^. Dermaseptin-S1 has shown potent activity against pathogenic fungi, Gram-positive and Gram-negative bacteria, protozoa, and genital pathogens. Its antimicrobial activity is mediated by the selective interaction of basic and amphipathic α-helix moieties with plasma membrane phospholipids, resulting in membrane permeabilization and lysis^30–33^.

In this study, we focused on assessing RetroMAD1's potential as a treatment for SARS-CoV-2 by targeting the key non-structural protein, 3-chymotrypsin-like protease (3CL^pro^), which plays a critical role in viral replication^34^. To evaluate RetroMAD1's effectiveness, we conducted molecular docking experiments, comparing it with reference drugs like Andrographolide, Ivermectin, and Remdesivir, all previously shown to inhibit SARS-CoV-2 3CL^pro^^35–37^. To delve deeper into the interaction between RetroMAD1 and 3CL^pro^, we performed extensive 100 ns molecular dynamics simulations to assess the dynamic stability of the RetroMAD1-3CL^pro^ complex. This computational analysis allowed us to examine the behaviour of the complex over time. Subsequently, we conducted *in vitro* experiments, including a 3CL protease inhibition assay, to validate our findings from the docking and molecular dynamics simulations. These assays helped confirm RetroMAD1's ability to inhibit 3CL^pro^ enzyme activity effectively. Lastly, we assessed RetroMAD1's capacity to prevent SARS-CoV-2 (Wuhan-Hu-1) infection using an in vitro EpiAirway Model, which consists of healthy human-derived tracheal/bronchial epithelial cells closely resembling the human respiratory tract. Our comprehensive study provides compelling evidence of RetroMAD1's potential as a promising candidate for SARS-CoV-2 treatment, achieved through the inhibition of 3CL^pro^ activity.

## Methods

### RetroMAD1 structure modelling

The complete amino acid sequence of RetroMAD1 was retrieved from the GenBank^®^ database (accession number: OQ714810) and served as the template for protein structure prediction using the default parameters of ColabFold in conjunction with Google Colab^38,39^. ColabFold replaced the AlphaFold2 homology search with an MMseqs2 search against both the UniRef and Environment sequence databases, using both paired and unpaired sequences^40,41^. The models were then visualized using YASARA Structure Version (20.10.4)^42^ and PyMOLv2.5 (PyMOL, 2020; https://pymol.org/2/)^43^. The predicted structure comprises the complete domains of retrocyclin -101, MAP30, and dermaseptin S-1, and this model was chosen for molecular docking (Fig. 1).

### Preparation of SARS-CoV-2 proteins and reference drugs

The crystal structure of SARS-CoV-2 3-chymotrypsin-like protease (3CL^pro^) (PDB ID: 6LU7) of Wuhan-Hu-1 variant are readily available in the Research Collaboratory for Structural Bioinformatics Protein Data Bank (RCSB PDB) (https://www.rcsb.org/) and were retrieved in the .pdb format. Swiss Model web server was used for repairing the missing residues within the structures^44^. All the crystal structures were prepared by removing existing co-crystallised ligands, water molecules and hetero atoms by PyMOLv2.5 (PyMOL, 2020; https://pymol.org/2/)^43^. The 3D structure of reference drugs in this study (Andrographolide, Ivermectin and Remdesivir) were retrieved from the PubChem database (https://pubchem.ncbi.nlm.nih.gov/)^45^. These ligands were converted into the .pdb format using the Open Babel tool^46^.

### Protein–protein docking

HDOCK server (http://hdock.phys.hust.edu.cn/), an online server which can predict protein–protein interaction through a hybrid algorithm of template-based docking^47^, was used to carry out protein–protein docking between RetroMAD1 as a ligand and SARS-CoV2 3CL^pro^ as a receptor. His-41 and Cys145 are the catalytic dyad that serve as the active site of 3CL^pro^^48^. Therefore, residues that located within 5.5 Å of Cys145 reported from previous study (Thr24, Thr25, Thr26, Leu27, His41, Met49, Tyr54, Phe140, Leu141, Asn142, Gln143, Ser144, Cys145, His163, His164, Met165, Glu166, Leu167, Pro168, His172, Asp187, Arg188, Gln189, Thr190, Ala191, Gln192)^49^ were defined as input active site residues to specify the binding area of the docking complex. Based on the procedure of HDOCK server, the top 100 docking results can be generated from each complex and the top 10 docking results can be visualized on result web page^47^. The RetroMAD1-3CL^pro^ complex with highest negative docking scores were assessed for the docking poses and visualized using PyMOLv2.5 (PyMOL, 2020; https://pymol.org/2/)^43^. Prodigy website (https://wenmr.science.uu.nl/prodigy/) was used to predict the values of binding affinity (ΔG, kcal/mol) at 25.0 °C.

### Molecular dynamic simulation (MDS)

The molecular dynamics of the RetroMAD1-3CL^pro^ complex generated from the HDOCK server were studied using the YASARA Structure software (YASARA Biosciences GmBH, Vienna, Austria), version 22.9.24. Subsequent to the preliminary structural preparations (structural cleanup and pH adjustment), the simulation was conducted within YASARA, using the AMBER14 force field integrated into the YASARA structure (macro: md_run.mcr), albeit with slight alterations. To elaborate, the Posisson-Boltzmann approach was the chosen technique for managing the electrostatic force to obtain electrostatic potential with implicit solvent and counter ions. A “simulation cubic cell” was set and inundated with water, maintaining a density of 0.997 g/mL and isobaric pressure at 298 K throughout the 100 ns simulation. To mimic neutral physiological environment, sodium chloride was introduced at a concentration of 0.9%. The distance between the protein and the cell boundary was set to 10 Å. The timestep was designated at 2.5 fs, with simulation snapshots taken at intervals of 250 ps. For a comprehensive analysis of energy and residue alterations throughout the simulation, the md_analyze.mcr and md_analyzebindenergy.mcr macros were employed. The findings, encompassing data on the root mean square deviation (RMSD), the root mean square fluctuation (RMSF), hydrogen bonds, radius of gyration, and the ligand binding energy (using Adaptive Poisson-Boltzmann Solver^50,51^ provide insights into the structural (dynamic) stability of the complex binding. In the YASARA binding energy function, the energy is calculated as the difference between the sum of potential and solvation energies of the separated compounds and the sum of potential and solvation energies of the complex in the AMBER14 force field. Thus, more positive energy (difference) means higher affinity.

### 3CL^pro^ inhibition assay

Test concentrations of RetroMAD1, ranging from 400 to 33.55 µg/mL, were prepared in the Dulbecco's Modified Eagle's Medium/Nutrient Mixture F-12 Ham (DMEM/F12) non-phenol red cell culture media (Gibco, Thermo Fisher Scientific, USA). The 3CL^pro^ inhibitor, feline antiviral drug (GC376) provided by BPS Bioscience, was employed as an inhibitor control. The effect of RetroMAD1 on inhibiting the activity of the 3CL^pro^ was assessed using the Untagged 3CL Protease Assay Kit (BPS Bioscience, Catalog # 78042-1) in accordance with the manufacturer’s instructions.

In brief, 3CL^pro^ was diluted in assay buffer (containing 1 mM DTT) to achieve a concentration of 0.5 ng/µl (15 ng per reaction). Subsequently, 30 µl of the diluted 3CL Protease enzyme solution was introduced into wells designated as “Positive Control,” “Inhibitor Control,” and “Test Sample.” In parallel, 30 µl of the Assay Buffer (containing 1 mM DTT) was added to the “Blank” well. Additionally, 10 µl of GC376 (500 µM) was pipetted into the wells labelled as the "Inhibitor Control." Furthermore, 10 µl of RetroMAD1 at specific diluted concentrations was added to each well marked as the "Test Sample." Finally, 10 µl of Assay Buffer with DTT was introduced into both the "Blank" and "Positive Control" wells.

The enzyme and inhibitors were subjected to preincubation for 30 min at room temperature with slow shaking. The reaction was initiated by the addition of 10 µl of the 3CL Protease substrate solution (200 µM) to each well. Subsequently, the sealed plate was incubated for 4 h at room temperature with slow shaking. Finally, fluorescence intensity was measured using an M3 multi-mode microplate reader (SpectraMax Molecular Devices) at an excitation wavelength of 360 nm and detection of emission at a wavelength of 460 nm.

### In vitro evaluation of antiviral efficiency and cytotoxicity in EpiAirway model

#### Compound

RetroMAD1 was diluted to test concentrations as follows: 400, 300, 200, 150, 100, 75, 50, and 37.5 µg/mL in the MatTek culture medium (AIR-100-MM). Remdesivir (MedChemExpress, cat# HY-104077) was tested in singlet wells at concentrations of 1, 0.1, 0.01, and 0.001 µg/mL as the positive control.

#### Virus

The USA-WA1/2020 strain of SARS-CoV-2 was passaged three times in Vero 76 cells to create the virus stock. The virus was diluted in AIR-100-MM medium before infection, resulting in a multiplicity of infection (MOI) of approximately 0.03 CCID50 per cell.

#### Determination of virus titers from each treated cell culture

Vero 76 cells (African green monkey (*Chlorocebus aethiops*) kidney; ATCC CRL-1587) were seeded in 96-well plates and grown overnight (37 °C, 5% CO_2_) to 90% confluence. Samples containing the virus were diluted in tenfold increments in the infection medium, and 200 µL of each dilution was transferred into respective wells of a 96-well microtiter plate. Four microwells were used for each dilution to determine 50% viral endpoints. After 7 days of incubation, each well was scored positive for the virus if any cytopathic effect (CPE) was observed compared to the uninfected control, and counts were confirmed for the endpoint on day 10. The virus dose that infected 50% of the cell cultures (CCID_50_ per 0.1 mL) was calculated using the Reed-Muench method^52^. The day 7 values are reported. Untreated, uninfected cells were used as cell controls.

#### Cell culture

The EpiAirway Model consists of normal, human-derived tracheal/bronchial epithelial (TBE) cells that have been cultured to form a multi-layered, highly differentiated model resembling the epithelial tissue of the respiratory tract. The cell cultures were custom-made by MatTek Life Sciences (https://www.mattek.com) (Ashland, MA) and arrived in kits with either 12- or 24-well inserts each. The TBE cells were grown on 6 mm mesh disks in transwell inserts. During transportation, the tissues were stabilized on a sheet of agarose, which was removed upon receipt. One insert was estimated to consist of approximately 1.2 × 106 cells. Kits of cell inserts (EpiAirway AIR-100, AIR-112) originated from a single donor, # 9831, a 23-year-old, healthy, non-smoking, Caucasian male. The cells have unique properties in forming layers, with the apical side exposed only to air, creating a mucin layer. Upon arrival, the cell transwell inserts were immediately transferred to individual wells of a 6-well plate according to the manufacturer's instructions, and 1 mL of MatTek’s proprietary culture medium (AIR-100- MM) was added to the basolateral side, while the apical side was exposed to a humidified 5% CO2 environment. The TBE cells were cultured at 37 °C for one day before the start of the experiment. After the 24-h equilibration period, the mucin layer, secreted from the apical side of the cells, was removed by washing with 400 µL pre-warmed 30 mM HEPES buffered saline solution 3X. Culture medium was replenished on the basal side following the wash steps.

#### Experimental design

Each compound treatment (120 µL) and virus (120 µL) were applied to the apical side, and compound treatment only was applied to the basal side (1 mL) for a 2-h incubation. As a virus control, three of the cell wells were treated with placebo (cell culture medium only). Following the 2-h infection, the apical medium was removed, and the basal side was replaced with fresh compound or medium. The cells were maintained at the air–liquid interface. On day 6, the medium was removed and discarded from the basal side. Virus released into the apical compartment of the tissues was harvested by the addition of 400 µL of culture medium pre-warmed at 37 °C. The contents were incubated for 30 min, mixed well, collected, thoroughly vortexed, and plated on Vero 76 cells for the virus yield reduction (VYR) titration assay. Triplicate and singlet wells were used for virus control and cell control, respectively.

#### Assay of cytotoxicity

Uninfected cells treated with each concentration of the test compound were run in parallel with the infected, treated wells. The cell cytotoxicity was determined under the microscope to check for any changes in cell appearance compared to the normal control cells and toxicity control cells that were run on the same plate. These changes may include enlargement, granularity, cells with ragged edges, a filmy appearance, rounding, detachment from the surface of the well, or other changes. These changes were designated as T (100% toxic), PVH (partially toxic–very heavy–80%), PH (partially toxic–heavy–60%), P (partially toxic–40%), Ps (partially toxic–slight–20%), or 0 (no toxicity–0%), depending on the degree of observed cytotoxicity.

### Data and statistical analysis

Data and statistical analysis were conducted using GraphPad Prism 9 software (GraphPad Software Inc., San Diego, CA, USA). The half maximal effective concentration (EC_50_) and half maximal inhibitory concentration (IC_50_) of RetroMAD1 were determined through non-linear regression of the inhibitor's concentration–response curve fit model. The EC90 was calculated using the GraphPad Prism 9 calculator, based on the EC50 value and hill slope obtained from non-linear regression analysis. Statistical analysis employed an unpaired t-test with Welch's correction, and the results are presented as the mean ± SD. P values less than 0.05 were considered statistically significant.

## Results

### Binding complexes of RetroMAD1 with SARS-CoV-2 3CL^pro^

**Figure 2:**
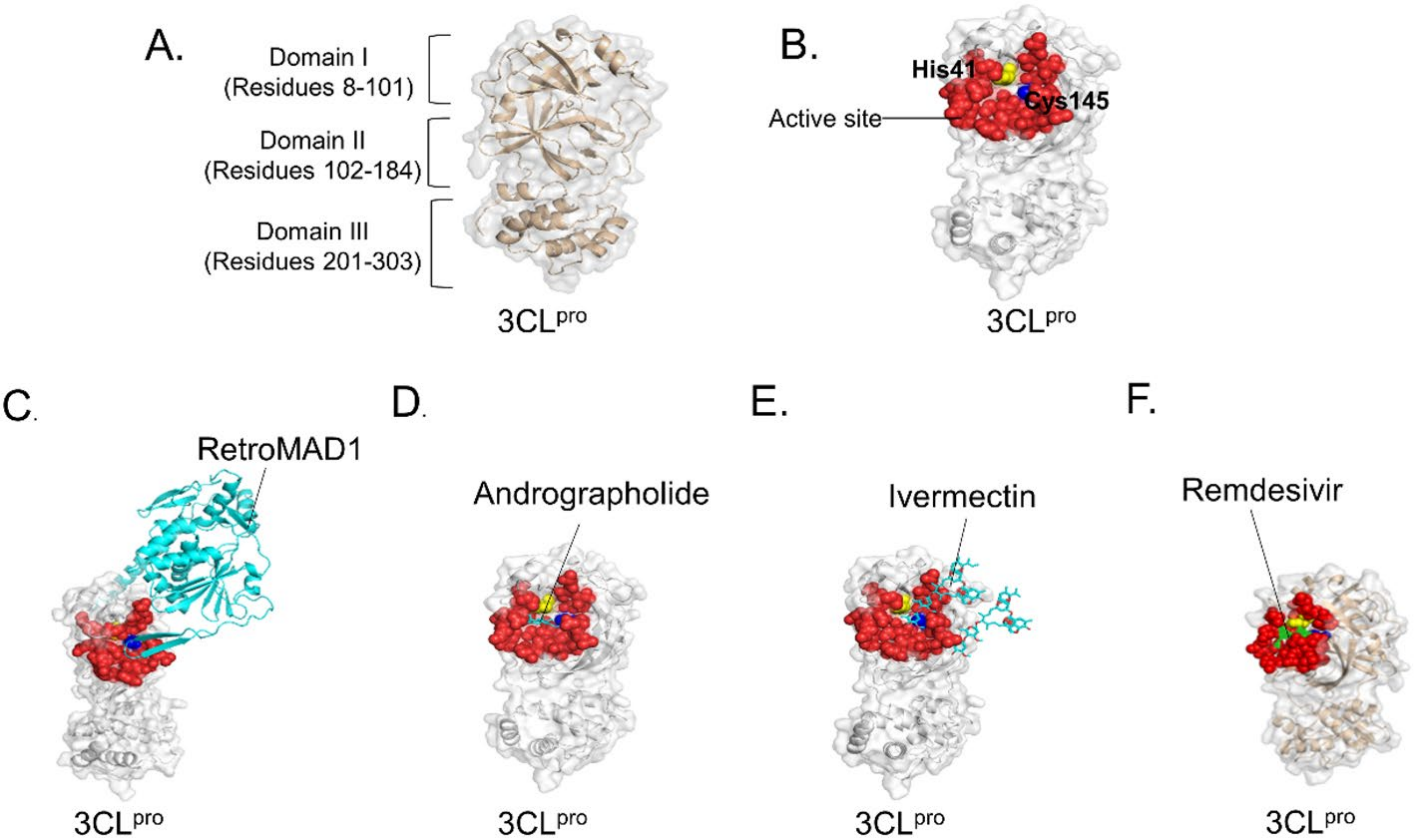
Binding of RetroMAD1 with 3CL^pro^. (**A**) Structure of SARS-CoV-2 3CL^pro^ monomer (PDB ID 6LU7). (**B**) Red colour indicates the active site of SARS-CoV-2 3CL^pro^ (Novak et al., 2021). The catalytic dyad, His41 and Cys145 depicted in yellow and dark blue respectively. The binding complex of 3CL^pro^ with (**C**) RetroMAD1, (**D**) Andrographolide, (**E**) Ivermectin and (**F**) Remdesivir. Figure was made by using Pymol.

The 3CL^pro^ enzyme is highly conserved among coronaviruses, with the sequence of 3CL^pro^ in SARS-CoV-2 sharing a 96% similarity with SARS-CoV^53^. It plays a crucial role in cleaving 11 distinct sites, generating various non-structural proteins. 3CL^pro^ is a homodimer, with each monomer consisting of 306 residues organized into three domains. Domains I (residues 8–101) and II (residues 102–184) feature a six-stranded antiparallel β-sheet structure, while domain III (residues 201–303) comprises five α-helices and is responsible for enzyme dimerization^53,54^ (Fig. 2A). The catalytic dyad, His41-Cys145, which acts as the binding site for the substrate, is located within a pocket between domains I and II^48^ (Fig. 2B).

Using the HDOCK server (http://hdock.phys.hust.edu.cn/), we assessed the potential of RetroMAD1 to inhibit 3CL^pro^ by conducting molecular docking of the RetroMAD1-3CL^pro^ complex. We utilized the SARS-CoV-2 3CL^pro^ model retrieved from the Protein Data Bank (PDB ID: 6LU7). Among the top 10 results, we selected the one with the highest negative scores for interaction with the catalytic site (His41-Cys145) and the other 25 important catalytic pocket residues (Thr24, Thr25, Thr26, Leu27, His41, Met49, Tyr54, Phe140, Leu141, Asn142, Gln143, Ser144, Cys145, His163, His164, Met165, Glu166, Leu167, Pro168, His172, Asp187, Arg188, Gln189, Thr190, Ala191, Gln192)^49^. Andrographolide, Ivermectin and Remdesivir, which have previously shown efficacy against 3CL^pro^^35–37^, were used as reference drugs. It was found that RetroMAD1 exhibits the strongest interaction with 3CL^pro^ (− 219.74), followed by Remdesivir (− 182.36), Ivermectin (-142.85) and Andrographolide (− 117.10) (Fig. 2C–F, Table 1). The docking complexes (RetroMAD1-3CL^pro^, Andrographolide-3CL^pro^, Ivermectin-3CL^pro^, and Remdesivir-3CL^pro^) underwent further evaluation for their binding affinities and dissociation constants using the PRODIGY server. Table 1 presents the binding affinities of the SARS-CoV-2 3CL^pro^ complexes with RetroMAD1, Andrographolide, Ivermectin and Remdesivir were − 12.3,  − 5.43, − 5.42 and − 5.53 kcal mol^−1^, respectively. RetroMAD1 exhibited a relatively higher binding affinity and bound to the His41-Cys145 catalytic dyad and other catalytic pocket residues.

**Table 1:** Docking scores of RetroMAD1 in complex with SARS-CoV-2 3CL^pro^.

Target	Drugs/Control	HDOCK docking Score	PRODIGY binding affinity, ΔG (kcal mol^−1^)	Binding residues
SARS-CoV-2 3CL^pro^	RetroMAD1	− 219.74	− 12.3	Gln19, Thr21, Gly23, **Thr24**, **Thr25**, **Thr26**, **Leu27**, Pro39, Arg40, **His41**, Val42, **Met49**, His64, Leu67, Gln69, Gly71, Asn72, Val73, Gln74, Asn119, Ser121, **Phe140**, **Leu141**, **Asn142**, **Ser144**, **Cys145**, Gly146, **His163**, **His164**, **Met165**, **Glu166**, **Leu167**, **His172**, **Gln189**
	Andrographolide	− 117.10	− 5.43	**His41, Met49, Phe140, Leu141, Asn142, Gly143, Ser144, Cys145, His163, His164, Met165, Glu166,** Val186**, Asp187, Arg188, Gln189, Thr190, Gln192**
	Ivermectin	− 142.85	− 5.42	**Thr25**, **Leu27**, Pro39, **His41**, Val42, Cys44, Thr45, Asp48, **Met49**, Leu50, **Tyr54**, **Leu141**, **Asn142**, **Gly143**, **Ser144**, **Cys145**, **His163**, **His164**, **Met165**, **Glu166**, **Leu167**, **Pro168**, Val171, **His172**, Ala173, Phe181, Val186, **Asp187**, **Arg188**, **Gln189**, **Thr190**, **Gln192**
	Remdesivir	− 182.36	− 5.53	**Thr25**, **Thr26**, **Leu27**, **His41**, **Met49**, **Leu141**, **Asn142**, **Gly143**, **Ser144**, **Cys145**, **His164**, **Met165**, **Glu166**, **Leu167**, **Pro168**, **Arg188**, **Gln189**, **Thr190**, **Gln192**

Interacting residues of 3CL^pro^ active sites are bolded.

### Molecular dynamic simulation

**Figure 3:**
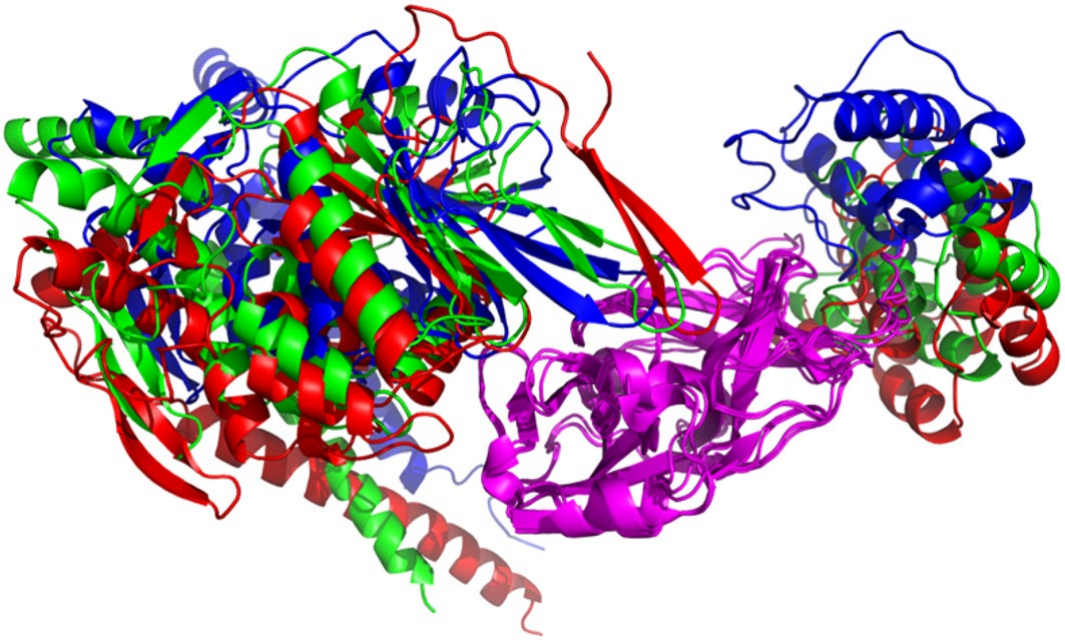
Initial YASARA MD snapshot, subsequent to YASARA's energy minimization, is depicted in red (0 ns), while the conformation following 100 ns of simulation is illustrated in blue, and the averaged structural snapshots is represented in green. The superimpositions were executed with respect to Domains I and II of the 3CL^pro^ using PyMOL Molecular Graphics System, Version 2.0 Schrödinger, LLC. It was discerned that interaction regions were consistently preserved throughout the computational simulation period.

To investigate the dynamic stability of the RetroMAD1-3CL^pro^ complex, molecular dynamics simulations were employed. Utilizing YASARA, the interaction of the complex was ascertained over a 100 ns simulation in an explicit solvation system, with respect to the starting structure (binding complexes from HDOCK server, 0 ns). The complex’s interaction was later computed using RMSD, RMSF, MM/PBSA, Hydrogen Bond, and Radius of Gyration values. The snapshots of the interaction of the complex were illustrated in Fig. 3; the initial (0 ns), average and last (100 ns).

**Figure 4:**
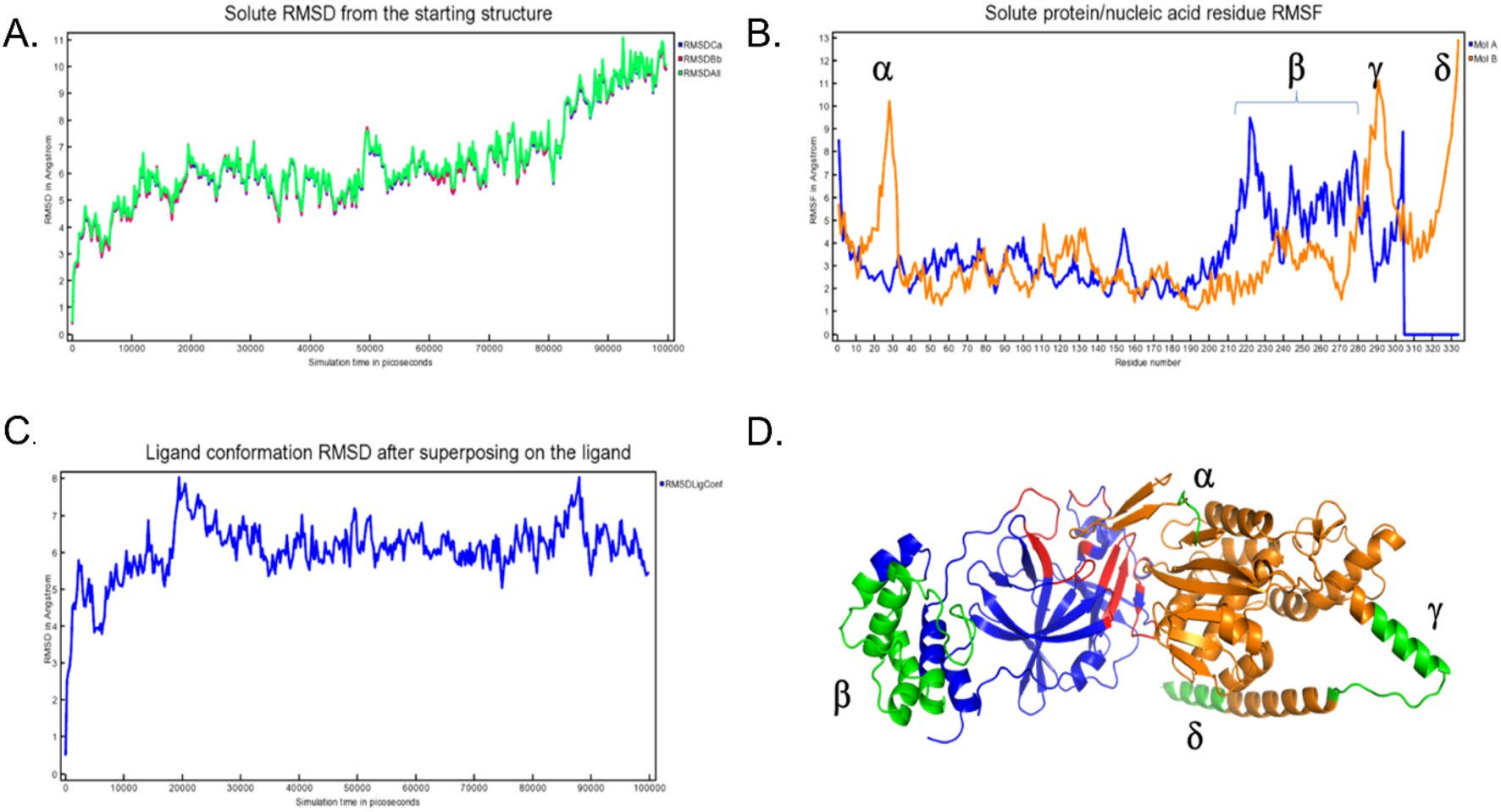
The molecular dynamics analysis of the 3CL^pro^-RetroMAD1 complex over 100 ns simulations. (**A**) The graph displays the root mean square deviation (RMSD) of the Cα atoms (blue), backbone atoms (red), and average RMSD (green). The trajectory reveals a stable binding interaction throughout the simulation, with fluctuation within permissible limit; (**B**) The root mean square fluctuation (RMSF) shows the per residue flexibility of 3CL_pro_ (blue) and RetroMAD1 (orange). Minor fluctuations at less 2 Å were observed for the contact residues; (**C**) RetroMAD1 conformation RMSD change compared to initial binding pose via HDOCK. The ligand shows little variation throughout the simulations; (**D**) Bound illustration of 3CL^pro^ (orange)-RetroMAD1 (blue) complex. Also highlighted in this cartoon representation in green, representing flexible structure and, in red, the 3CL_pro_ residues bearing less than 5 Å contact distance to RetroMAD1. The insets in Subfigures (**B**) and (**D**) show the relationships between the high RMSF residues and the structural locality of the high RMSF regions within the bound structures: Residues 18–35 (α), 280–300 (γ) and 315–335 (δ) of RetroMAD1 and residues 210–280 (β) of 3CL^pro^ display highest flexibility relative to the overall binding dynamics of the complex.

The RMSD trajectory (Fig. 4A), revealed an initial destabilization within the first 10 ns subsequently settling into a stable structural comportment with RMSD values oscillating between 0.4 to 0.8 nm, with respect to the starting structure. A noticeable spike in RMSD value at 80 ns was observed and later stabilized at an average oscillating value of 0.2 nm for the remainder of the simulation. At the residue level, the root square fluctuation (RMSF) was analysed to discern the flexibility of protein residues throughout the simulation (Fig. 4B)—the RMSF per solute residue was calculated from the average RMSF of the atoms constituting the residue. Notably, it was observed that minor fluctuations at less 0.2 nm were observed for the contact residues with major fluctuations exceeding 1 nm, were attributed to residues 18–35, 280–300 and 315–335 of RetroMAD1 and residues 210–280 of 3CL^pro^. Fortunately, these regions were not implicated in RetroMAD1-3CL^pro^ contact sites thus might suggest structural integrity or flexibility that was not attributed directly from the RetroMAD1-3CL^pro^ complex formation (Fig. 4C,D).

**Figure 5:**
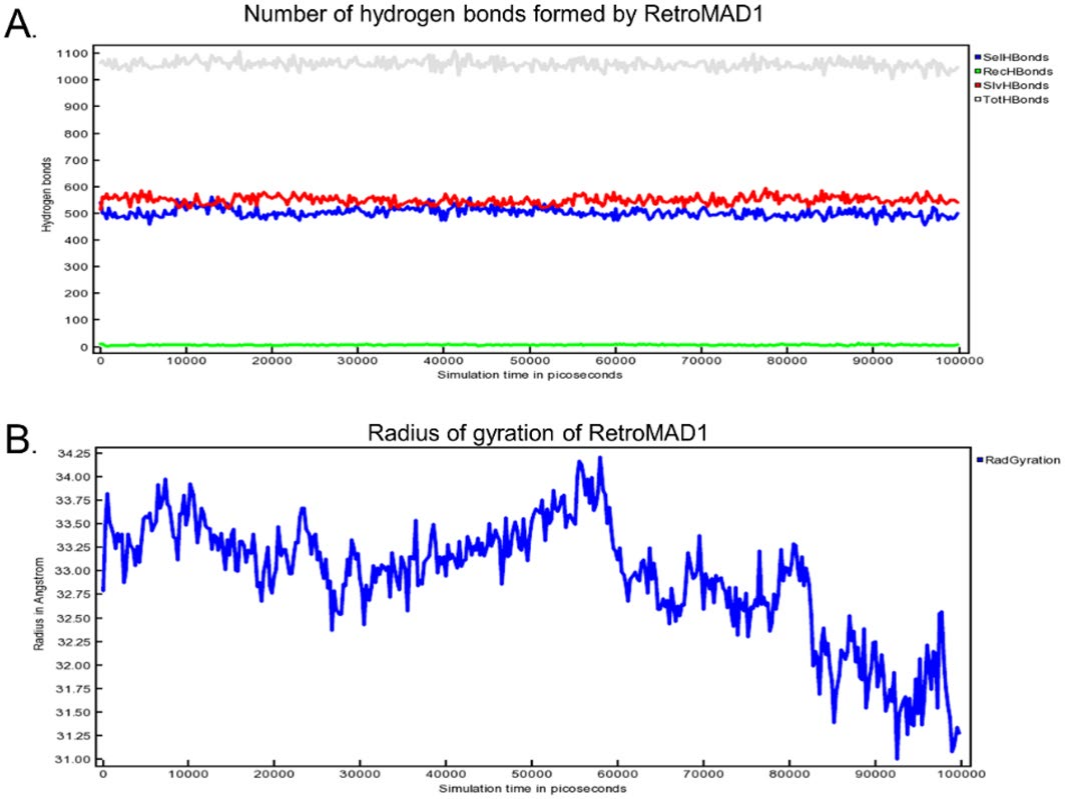
Analysis of interaction via hydrogen bonding counts and radius of gyration. (**A**) The hydrogen bonding is categorized as follows: intrinsic molecular hydrogen bonds are denoted in blue, bonds formed with the adjacent 3CL^pro^ are in green, and those engaged with the aqueous environment are represented in red. Cumulative hydrogen bonds is depicted in gray. (**B**) The radius of gyration of RetroMAD1-3CL^pro^ complex corresponds to 100 ns molecular dynamics simulation at 298 K.

The average Molecular Mechanics Poisson-Boltzmann Surface Area (MM/PBSA) was calculated to be at -110.460 kJ/mol, with the best interaction during 43.25 ns at 604.556 kJ/mol. Additionally, the hydrogen bond count remains proximate to 1100 (Fig. 5A), indicating a consistent interaction while data from radius of gyration suggests the complex exhibits consistent stability with limited structural movement, with less than 0.3 nm throughout the span of the 100 ns simulation (Fig. 5B). Additional (raw) data on MM/PBSA, hydrogen bonds analysis and radius of gyration can be found at the Supplementary Information S1.

### RetroMAD1 inhibits 3CL^pro^ enzyme activity

**Figure 6:**
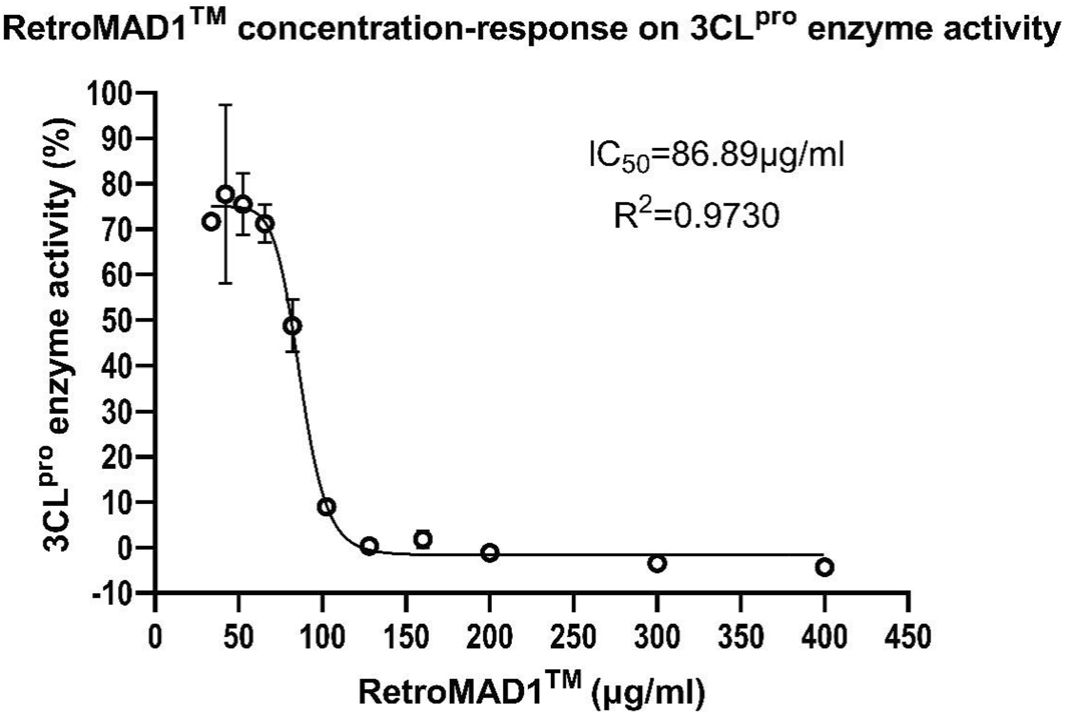
Concentration–response curve for determining the half-maximal inhibitory concentration (IC_50_) of RetroMAD1 against 3CL protease enzyme activity, assayed using the Untagged 3CL Protease (SARS-CoV-2) Assay Kit (BPS Bioscience #78,042). The calculated IC_50_ value for RetroMAD1, inhibiting 3CL^pro^ activity, was found to be 86.89 μg/mL.

To better understand how RetroMAD1 inhibits SARS-CoV-2 replication, we conducted a study to examine its inhibitory activity and compared it to GC376, a protease inhibitor that targets the 3CL^pro^ enzyme^55^ (Table 2). We performed statistical analysis using unpaired t-tests with Welch's correction to assess the inhibition of 3CL^pro^ enzyme activity by RetroMAD1 at concentrations of 33.55, 41.94, 52.43, 65.54, 81.92, 102.4, 128, 160, 200, 300, and 400 μg/mL) against GC376 at a concentration of 500 μM (Table 2). The analysis revealed that there were no any significant differences in the inhibition of 3CL^pro^ enzyme activity between RetroMAD1 at concentration above 128 μg/mL (Table 2, bolded) and GC376 at concentration of 500 μM (Table 2, *italicized*). We also generated a concentration–response curve for RetroMAD1's inhibition of 3CL^pro^ activity, which revealed an IC_50_ of 86.89 μg/mL (Fig. 6).

**Table 2:** 3CL^pro^ enzyme Inhibition by RetroMAD1.

Test compounds	Concentration	3CL^pro^ inhibition (%)	SD	Unpaired t test with Welch's correction, p-value
GC376	*500 μM*	*101.35*	1.08	NA
RetroMAD1	33.55 μg/mL	28.35	1.60	< 0.0001*
	41.94 μg/mL	22.29	19.65	0.0197*
	52.43 μg/mL	24.48	6.75	0.0021*
	65.54 μg/mL	28.79	4.14	0.0006*
	81.92 μg/mL	51.29	5.72	0.0033*
	102.4 μg/mL	91.03	1.59	0.0013*
	**128 μg/mL**	**99.63**	1.47	**0.1842**
	**160 μg/mL**	**98.19**	1.86	**0.0787**
	**200 μg/mL**	**101.13**	1.12	**0.8173**
	**300 μg/mL**	**103.44**	0.60	**0.0581**
	**400 μg/mL**	**104.30**	1.47	**0.0531**

The data presented in this study represent the mean values obtained from three biological replicates. An unpaired t-test with Welch's correction was employed to evaluate the statistical significance of differences between GC376 and RetroMAD1 at each tested concentration. Asterisks (*) indicates significant threshold of P<0.05. NA is not applicable.

Bolds are concentrations of RetroMAD1 with no significant difference compared to the tested concentration of GC376.

### RetroMAD1 inhibits SARS-CoV-2 infection

To assess RetroMAD1's antiviral properties in a model that closely resembles human cell infection, we utilized the EpiAirway Model. This model involves the cultivation of normal human-derived tracheal/bronchial epithelial (TBE) cells to create a multi-layered, highly differentiated structure that closely mimics respiratory epithelial tissue^56^. Our results obtained using the EpiAirway Model did not reveal any microscopic signs of cytotoxicity at any of the tested RetroMAD1 doses (microscopy images are not shown).

To assess the effectiveness of RetroMAD1 in preventing SARS-CoV-2 infection in human-derived TBE cells, we conducted an evaluation of its inhibitory impact on SARS-CoV-2 at various concentrations, comparing it to the efficacy of Remdesivir (see Table 3). Subsequently, in a viral yield reduction assay conducted in Vero-76 cells using virus-containing supernatant, RetroMAD1 exhibited efficient inhibition of SARS-CoV-2 replication at concentrations of 400 μg/mL and 300 μg/mL, resulting in log_10_ CCID_50_ values of 2.67 and 2.5, respectively (Table 3, *italicized*). In the absence of any antiviral compound, cells exposed to the infection media produced a log_10_ CCID_50_ value of 4.4.

**Table 3:** Antiviral efficiency of RetroMAD1 against SARS-CoV-2.

Test compounds	Concentration (µg/mL)	^a^Log_10_ CCID_50_ virus per 0.2 mL	^b^EC_90_ (µg/mL)
RetroMAD1	*400*	*2.5*	157
	*300*	*2.67*	
	200	3.5	
	150	3.67	
	100	3.67	
	75	4	
	50	4.3	
	37.5	4	
Remdesivir	1	1.67	0.006
	**0.1**	**2.67**	
	0.01	3.5	
	0.001	3.67	
Virus control	NA	4.4	NA

The data presented here represent the mean values obtained from three biological replicates. Each well was considered positive for virus presence if any Cytopathic Effect (CPE) was observed in comparison to the uninfected control. Assessment of Vero-76 cells was conducted on day 7 and confirmed on day 10.

*NA* is not applicable.

The concentrations of RetroMAD1 that demonstrated equally effective inhibition of SARS-CoV-2 replication as Remdesivir at 0.1 µg/mL are in *italics*.

^a^Titer results originate from the virus yield reduction assay.

^b^EC_90_ denotes the 90% effective concentration, which is the concentration required to reduce virus yield by 1 − log_10_, as determined through regression analysis.

Overall, the data from this physiologically relevant human bronchial cell model indicate that RetroMAD1 effectively reduced viral replication by 41.2 percent. This reduction is equivalent to Remdesivir's ability to suppress SARS-CoV-2 replication at a concentration of 0.1 μg/mL (Table 3, bolded). An analysis of the concentration–response relationship yielded an EC_90_ of 157 μg/mL for RetroMAD1 to reduce SARS-CoV-2 infectivity by 1 − log_10_.

## Discussion

The ongoing COVID-19 pandemic presents a global challenge, emphasizing the urgent need for the development of effective vaccines and treatments capable of addressing evolving viral variants. The effectiveness of current COVID-19 vaccines designed for SARS-CoV-2 may diminish due to frequent mutations, leading to changes in its antigenic properties. Despite the widespread use of highly effective vaccines that can prevent severe disease and hospitalization, they have not been entirely successful in curbing the virus's transmission in many countries^57^. Consequently, it is crucial to persistently seek improved or repurposed antiviral drugs. In this context, peptide-based therapeutics offer distinct advantages over antibody-based drugs and small molecule drugs, primarily due to their specificity and reduced potential to trigger immune responses^58,59^. Furthermore, peptide drugs are easier to manufacture on a large scale, resulting in reduced production costs and faster manufacturing processes^60^.

RetroMAD1, designed to express three anti-viral peptide fusion proteins, offers a compelling direction for research. Since 2020, RetroMAD1 has been globally available for therapeutic intervention against FeLV and FCoV infections in *Catus domesticus* (indoor cats)^5^. Drawing on the structural congruence between FCoV and SARS-CoV-2^61^, our study delves into the prospective efficacy of RetroMAD1 against the 3CL^pro^ of SARS-CoV-2. We embarked on this exploration using molecular docking and advanced it with rigorous evaluations, including 100 ns molecular dynamics simulations to discern the nuanced interplay between RetroMAD1 and 3CL^pro^. Additionally, a series of in vitro inhibitory assays further illuminated the potential of RetroMAD1 in countering COVID.

SARS-CoV-2 3CL^pro^ is a non-structural protein responsible for cleaving at least 11 sites on large viral polyproteins that are essential for replication and transcription^62,63^. Consequently, inhibiting the activity of 3CL^pro^ could disrupt the SARS-CoV-2 viral replication, making it a crucial target for COVID-19 treatment. In line with this, numerous research groups have focused on 3CL^pro^ as they seek effective treatment for SARS-CoV-2 infection, leveraging repurposed antiviral drug such as Ribavirin, Remdesivir, Favipiravir, and Lopinavir, which are designed to impede viral replication^48,64–66^. Our docking study indicates that RetroMAD1 achieves a better docking score against 3CL^pro^ compared to Andrographolide, Ivermectin and Remdesivir, employing similar docking parameters (Table 1). It was also noticed that the molecular docking results demonstrated that RetroMAD1 interacts with the active site with retrocyclin binds to the active site and obstructing the catalytic dyad His41 and Cys145 of 3CL^pro^ (Fig. 2C). Although retrocyclin appears to be the primary component of RetroMAD1 interacting with SARS-CoV-2 3CL^pro^, the other two components, dermaseptin-S1 and MAP30 may also be able to effectively target other proteins of SARS-CoV-2. In fact, a current study revealed that MAP30 alone can inhibit SARS-CoV-2 replication in human lung cancer cells with relatively low cytotoxicity^67^. Additionally, in silico studies have also showed that dermaseptin or dermaseptin-based peptide exhibit high affinity for the Spike protein of SARS-CoV-2^68,69^. Hence, RetroMAD1 may possess broad-spectrum antiviral capabilities against various viral proteins. Further investigations are warranted to explore RetroMAD1’s effectiveness on other viral proteins that play an important role in viral replication, thereby elucidating its specificity for COVID-19 treatment.

To substantiate the docking studies, molecular dynamics (MD) trajectory analysis simulations were completed using Yasara Structure version 23.5.19.W.64 over a 100 ns with 400 snapshots, using AMBER14 force field. A steady-state NpT molecular dynamics simulation was utilized to validate the predicted binding between the RetroMAD1 and 3CL^pro^ and confirm the structural rigidity of their complex over 100 ns. The total potential energy of the system is available as Supplementary Information S2. As outlined in past work by Law and colleagues (2005), tracking deviations in the C_alpha_ backbone structure from the initial to final conformations using root mean square deviation (RMSD) analysis provides insights into binding drift^70^. Our RMSD trajectory results revealed an overall stability of the protein complex, especially within the first 80 ns. More pronounced conformational shifts arose after this period and later stabilized until the end of the simulation. Intriguingly, the RMSD superimposition assessment of RetroMAD1 illustrates that the ligand's conformation exhibits considerable constancy throughout the investigation, as visualized in Fig. 4C. Further analysis of residues of contact via superimposition of binding poses did not show a major shift or disengagement between the RetroMAD1-3CL^pro^ complex (Fig. 3). At the residual level, the Root Mean Square Fluctuation (RMSF) shows average flexibility of residue throughout the simulation. Through the elucidation of residue-level flexibility analysis, our findings accentuate that the predominant fluctuations arise mainly from non-binding residues within RetroMAD1, (Fig. 4B,D). An array of tables and advanced plots including the ‘dynamic cross-correlation matrix’ were, likewise, generated. Results from these additional plots, together, re-enforced the stability of the complex is available as Supplementary Information S3.

Subsequently, we conducted in vitro experiments, including a 3CL protease inhibitory assay and the use of human-derived TBE cells through EpiAirway Model, which provided additional compelling evidence of RetroMAD1's effectiveness in preventing SARS-CoV-2 infection. Remarkably, RetroMAD1 exhibited 101.13% to 104.30% inhibition against 3CL^pro^ enzyme activity at concentrations ranging from 200 to 400 μg/mL. This inhibition is equivalent to the inhibitory activity of GC376 at 500 μM, resulting in 101.35% inhibition (Table 2). Notably, our in vitro results (Table 3) using human-derived TBE cells also indicated that RetroMAD1 exhibited substantial inhibitory activity against SARS-CoV-2 (Wuhan-Hu-1) at a concentration of 300 to 400 μg/mL. Importantly, this level of effectiveness was on par with that of Remdesivir, an FDA-approved drug for COVID-19 treatment, which demonstrated its efficacy at 0.1 μg/mL^71^. Moreover, it's worth noting that no evidence of cytotoxicity to human-derived TBE cells was observed at this concentration. However, further investigations are warranted to determine the highest doses that may have detrimental effects on human cells.

It's worth noting that Remdesivir has shown limited effectiveness in treating hospitalized COVID-19 patients, as evidenced by its impact on overall mortality, the need for ventilation, and the duration of hospitalization^72^. This limited effectiveness may be attributed to the potential development of resistance by evolving SARS-CoV-2 variants^73–75^. The possibility of such resistance was reported by Agostini et al. (2018), where the EC_50_ of Remdesivir had to be increased from 0.01 to 0.06 μM in evolved coronavirus cultures carrying mutations within non-structural proteins 12 (nsp12) of the viral RNA-dependent RNA polymerase (RdRp)^76^. The constant evolution of SARS-CoV-2 may necessitate frequent increases in dosage or prolonged treatment with Remdesivir. Additionally, exposure to Remdesivir in concentrations ranging from 1 to 8 μM has been shown to lead to alterations in gene expression profiles and morphology^77^.

With this in mind, we propose a strategy for future studies involving the combination of repurposed drugs such as RetroMAD1 with Remdesivir. This strategy leverages Remdesivir's dual antiviral action, targeting both the RNA-dependent RNA polymerase (RdRp, also known as nsp12) and 3CL^pro^^78^. By combining Remdesivir with RetroMAD1, our aim is to potentially enhance their antiviral effectiveness. RetroMAD1's unique mechanism of action, attributed to the co-expression of three different peptides within RetroMAD1, equips it with the ability to combat a wide range of viruses. This distinctive feature makes RetroMAD1 less susceptible to the development of viral resistance, positioning it as a promising candidate for combination therapy alongside Remdesivir and other antiviral drugs. This strategy has the potential to reduce the risk of drug resistance while enhancing therapeutic efficacy. It should be further explored through in-vivo studies, followed by clinical trials^79^. It's worth noting that RetroMAD1 can be administered orally, which presents a significant advantage. This attribute could potentially make RetroMAD1 a preferred treatment option for mild-to-moderate COVID-19 cases in adults at high risk for severe diseases, including hospitalization and mortality.

While this study did not demonstrate RetroMAD1's effectiveness against any Omicron subvariants, which is a limitation of this study, it is hypothesized that there may be no 3CL^pro^ mutations that confer resistance to RetroMAD1. This hypothesis is grounded in the limited reports of resistance to 3CL^pro^ inhibitors^80^. Furthermore, recent research^81^ has shown that in vitro results indicate that GS-441524, Remdesivir, EIDD-1931, Molnupiravir, and Nirmatrelvir retain their activity against all Variants of Concern (VoCs), including Omicron. This suggests that RetroMAD1 may also have the capability to maintain its activity against future subvariants of Omicron. These findings provide a promising outlook for the potential effectiveness of RetroMAD1 in addressing emerging Omicron subvariants, although further research and testing are required to confirm this hypothesis.

Consequently, it is advisable for RetroMAD1 to undergo in vivo non-human primate challenge studies, followed by clinical trials, as a promising antiviral candidate for addressing infections caused by various SAR-CoV-2 variants, including Omicron subvariants. In-vivo studies are necessary to explore the limitations and strengths of RetroMAD1 in combating current SARS-CoV-2 variants before progressing to human clinical trials. The use of RetroMAD1, either alone or in combination with FDA-approved repurposed drugs, requires further validation through high-quality evidence and additional validation studies.

## Conclusion

SARS-CoV-2 3CL^pro^ represents a crucial target for COVID-19 treatment, and our study underwent rigorous validation, encompassing docking, molecular dynamics simulations, enzyme inhibitory assays, and EpiAirway Model testing. During the docking analysis, RetroMAD1 was compared to reference drugs (Remdesivir, Ivermectin, and Andrographolide), and RetroMAD1 exhibited a strong interaction with 3CL^pro^. Its binding affinity had the lowest negative binding energy (− 12.3 kcal/mol), exceeding that of Remdesivir, Ivermectin, and Andrographolide (− 5.53 kcal/mol, − 5.42 kcal/mol, − 5.43 kcal/mol). Exploring the dynamic stability of the RetroMAD1-3CL^pro^ complex through 100 ns molecular dynamics simulations, the RMSD trajectory showed an initial destabilization phase followed by stable structural behavior. Minor fluctuations in root mean square fluctuation (RMSF) were observed in specific residue regions rather than between the contact points of the complex. Additionally, the MM/PBSA analysis indicated favorable interaction energies during the simulation, consistently accompanied by hydrogen bond formation. Radius of gyration data further suggested limited structural movement, confirming overall complex stability. In vitro tests on human-derived TBE cells using the EpiAirway Model and 3CL protease inhibition activity supported the potential of RetroMAD1 as an antiviral therapy for COVID-19. However, further clinical research and rigorous trials are necessary to confirm its efficacy and safety for clinical application.

### Supplementary Information

Supplementary Information 1.

Supplementary Information 2.

Supplementary Information 3.

## Data Availability

The datasets generated and/or analysed during the current study are available in the GenBank repository, BankIt2688861 RetroMAD1 OQ714810.
