# Supplementary material for: Inhibition of SARS-CoV-2 3CL protease by the anti-viral chimeric protein RetroMAD1
Source: Sci Rep. 2023 Nov 17;13:20178. doi: 10.1038/s41598-023-47511-z (PMC10656507; doi:10.1038/s41598-023-47511-z)

Inhibition of SARS-CoV-2 3CL Protease by the Anti-viral Chimeric Protein RetroMAD1

Lee-Chin Chan[1,2ⴕ], Aini Syahida Mat Yassim[1,2,3ⴕ*], Abdullah Al Hadi Ahmad Fuaad[4], Thean Chor Leow[5,6,7], Suriana Sabri[5,6], Radin Shafierul Radin Yahaya[5], Awang Muhammad Sagaf Abu Bakar[8*]

[1] Biovalence Sdn. Bhd., 22, Jalan SS 25/34, Taman Mayang, 47301 Petaling Jaya, Selangor, Malaysia.
[2] Biovalence Technologies Pte. Ltd., #06-307 The Plaza, 7500A Beach Road, Singapore 199591.
[3] School of Health Science, Universiti Sains Malaysia, 16150 Kubang Kerian, Kelantan, Malaysia.
[4] Centre of Fundamental and Frontier Sciences in Self-Assembly (FSSA), Department of Chemistry, Faculty of Science, Universiti Malaya, 50603 Kuala Lumpur, Malaysia.
[5] Department of Microbiology, Faculty of Biotechnology and Biomolecular Sciences, Universiti Putra Malaysia, 43400 UPM Serdang, Selangor, Malaysia.
[6] Enzyme and Microbial Technology Research Center, Faculty of Biotechnology and Biomolecular Sciences, Universiti Putra Malaysia, 43400 UPM Serdang, Selangor, Malaysia.
[7] Institute of Bioscience, Universiti Putra Malaysia, 43400 UPM Serdang, Selangor, Malaysia.
[8]Jabatan Perkhidmatan Veterinar Sabah, Aras 3, Blok B, Wisma Pertanian Sabah, Jalan Tasik, Luyang (Off Jln Maktab Gaya), Beg Berkunci 2051, 88999 Kota Kinabalu, Sabah, Malaysia.

ⴕ These authors contributed equally to this work.

*Corresponding authors: syahida@biovalence.com (ASMY), awangsagaf@hotmail.com (AMSAB)

Supplementary Information, S2: Total potential energy of the system.

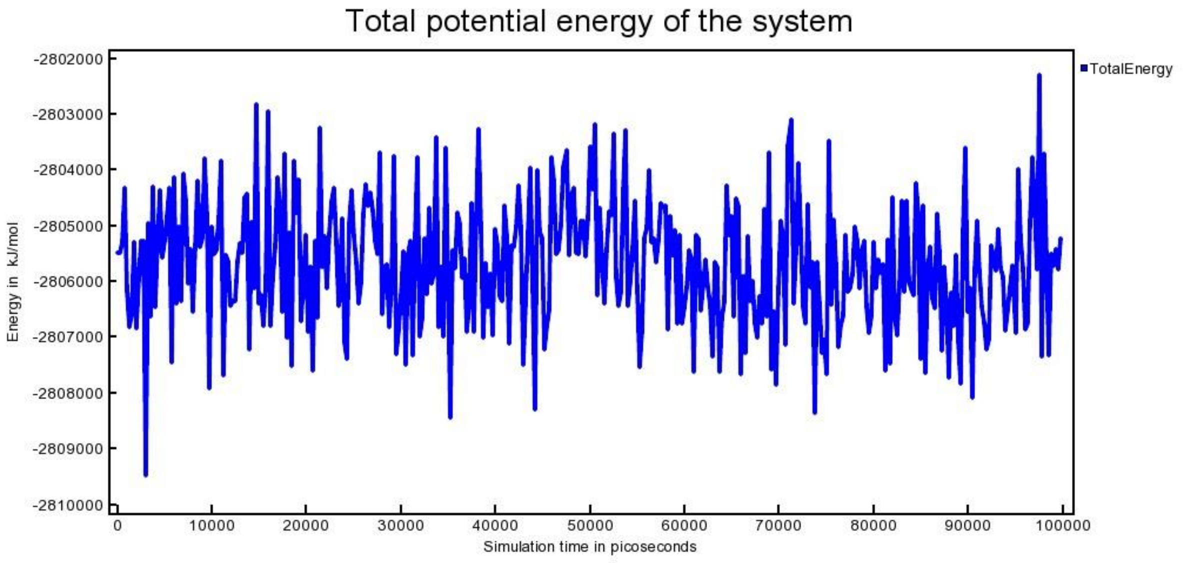

Supplement: Supplementary file 2 — Supplementary Information 2. [file 41598_2023_47511_MOESM2_ESM.pdf]
